# High-altitude immune remodeling in children with *Mycoplasma pneumoniae* pneumonia: a multi-center transcriptome study

**DOI:** 10.3389/fimmu.2025.1704739

**Published:** 2025-12-16

**Authors:** Yingying Luo, Jing Hou, Weifen Lu, Haixia Cao, Erwa Hao, Yaogang Zhang, Feng Liu, Yifan Zhu, Yanyan Ma

**Affiliations:** 1 Central Laboratory, Qinghai University Affiliated Hospital (Clinical Medical College), Xining, China; 2 Department of Respiratory Medicine, Children's Hospital of Nanjing Medical University, Nanjing, China; 3 Pediatrics, Qinghai University Affiliated Hospital (Clinical Medical College), Xining, China

**Keywords:** *Mycoplasma pneumoniae pneumonia*, children, altitude, immune response, transcriptome

## Abstract

**Introduction:**

A global outbreak of *Mycoplasma pneumoniae* pneumonia (MPP) emerged following the Corona Virus Disease 2019 (COVID-19) pandemic. Different altitudes significantly impact community-acquired pneumonia (CAP). Whether there are differences in the immune responses of children with MPP at different altitudes is unknown.

**Methods:**

This study collected bronchoalveolar lavage fluid (BALF) from children with MPP in Nanjing (altitude, 20 m) or Qinghai (altitude, 4,058.40 m) for transcriptome sequencing analysis to investigate pulmonary immune responses in children with MPP at different altitudes.

**Results:**

Children with refractory *Mycoplasma pneumoniae* pneumonia (RMPP) in the plateau group showed milder clinical manifestations, with shorter duration of fever before admission, lower levels of serum tissue injury markers, and lower incidences of atelectasis. Neutrophils and T cells in the lungs of the plain group were activated, along with the activation of the NOD-like receptor signaling pathway or the inhibition of the PI3K–Akt signaling pathway. The immune cells of children with MPP in the plateau group showed adaptive changes, which manifested as increased M2 polarization of macrophages, CD8^+^ T cells, and B cells, and decreased neutrophils and CD4^+^ T cells.

**Conclusion:**

Excessive activation of the pulmonary immune response in children with MPP of the plain region may be related to the NOD-like receptor signaling pathway and PI3K–Akt signaling pathway. Adaptive changes in immune cells occurred in children with MPP of the plateau region.

## 1 Introduction

*Mycoplasma pneumoniae* pneumonia (MPP) is one of the most common types of community-acquired pneumonia (CAP) in children, with an epidemic peak occurring approximately every 3–7 years worldwide (1, 2). MPP is generally regarded as a self-limiting disease, but some children develop refractory *M. pneumoniae* pneumonia (RMPP), characterized by persistent fever, progression of pulmonary imaging findings, and extrapulmonary complications after 7 days or more of regular treatment with macrolide antibiotics (3). After the COVID-19 pandemic, the global incidence of MPP has significantly increased. Meanwhile, RMPP has been observed in a large number of children, seriously threatening the physical and mental health of children (4–7).

Plateau regions, whose altitude typically exceeds 2,500 m, present unique physiological stressors, including hypobaric hypoxia, intense ultraviolet radiation, and extremely low temperatures. Long-term residence in high-altitude regions may lead to weakened immune responses. Residents of high-altitude regions exhibit decreased levels of circulating B cells, CD4^+^ T cells, dendritic cells, and cytokines [such as growth-regulated oncogene alpha (GROa), macrophage inflammatory protein-1 beta (MIP-1β), and interleukin-8 (IL-8)], alongside increased numbers of CD8^+^ T cells and natural killer (NK) cells (8–10). Chronic high-altitude exposure significantly reduces the number and proliferation of splenic T cells in mice, where the number of CD4^+^ T cells is significantly lower than that of CD8^+^ T cells, accompanied by decreased levels of IFN-γ (11). In addition, the concentrations of secretory immunoglobulin A (sIgA) decreased in athletes undergoing mid-altitude living and low-altitude training (12). High-altitude climate therapy (>1,500 m) can alleviate type 2 immune responses and the activation of T cells and monocytes in patients with allergic asthma (13–15). In Ecuador and Pakistan, the incidence of pneumonia among children in high-altitude regions is significantly higher than that in low-altitude regions (16, 17). Physiological adaptation to hypoxia may alleviate the severity of pneumonia (18). However, it is still unclear whether there are differences in the immune responses of children with MPP at different altitudes.

Bronchoalveolar lavage fluid (BALF) from children with MPP in Nanjing (altitude, 20 m) or Qinghai (altitude, 4,058.40 m) was collected for transcriptome sequencing analysis. This study revealed excessive pulmonary immune responses in children with MPP of the plain region, manifested as the activation of neutrophils and T cells, which may be related to the activation of the NOD-like receptor signaling pathway or the inhibition of the PI3K–Akt signaling pathway. Additionally, adaptive changes occurred in the immune cells of children with MPP in the plateau region, characterized by increased M2 polarization of macrophages, CD8^+^ T cells, and B cells, and decreased neutrophils and CD4^+^ T cells. This study contributes to understanding the impact of altitude on the pathogenesis of MPP to facilitate the precise treatment of MPP in different regions.

## 2 Methods

### 2.1 Study participants

This study prospectively included 65 children with MPP who were admitted to the Children’s Hospital of Nanjing Medical University (from January to December 2021) or Qinghai University Affiliated Hospital (from June 2023 to October 2024).

The inclusion criteria for children enrolled were as follows: 1) meeting the diagnostic criteria for CAP (19); 2) consistent with MP infection: positive MP nucleic acid test of nasopharyngeal aspirates, sputum, or BALF; and 3) needing fiberoptic bronchoscopy: after appropriate management and treatment with macrolide antibiotics, but still accompanied by poor clinical manifestations or pulmonary imaging.

Children were excluded if 1) any other pathogens were detected in their throat swabs, nasopharyngeal aspirates, sputum, BALF, or blood via culture, viral antigen detection assays, nucleic acid detection of respiratory pathogens, or T-cell spot tests (T-SPOTs); 2) they had underlying diseases at admission, such as chronic respiratory diseases, autoimmune diseases, cardiovascular diseases, or tumors; 3) they had a history of pneumonia within 28 days before admission; and 4) they did not agree to participate in this clinical study.

The defining characteristics of RMPP are persistent fever, worsening pulmonary imaging findings, or extrapulmonary complications after 7 days or more of regular treatment with macrolide antibiotics.

### 2.2 Clinical data and sample collection

Clinical data such as duration of fever before admission, hospitalization days, blood routine test results, blood biochemistry, coagulation function, and chest imaging were collected from enrolled children. Fiberoptic bronchoscopy was performed under a combination of intravenous and inhalation anesthesia. BALF was collected from the most severely affected lobe and immediately stored at −80 °C for sequencing.

### 2.3 Nucleic acid extraction, library preparation, and sequencing

Total RNA was extracted, and ribosomal RNA was removed. cDNA was generated using reverse transcriptase and dNTPs (Thermo Fisher, Waltham, Massachusetts, USA). Libraries were constructed for the cDNA samples using a Nextera XT DNA Library Prep Kit (Illumina, San Diego, CA, USA). Library pools were then loaded onto an Illumina Nextseq CN500 sequencer for 75 cycles of single-end sequencing to generate approximately 20 million reads for each library (20).

### 2.4 Transcriptome analysis

The read count normalization and differential expression analyses were performed using the DESeq2 package. The clusterProfiler package was used for Gene Ontology (GO) and Kyoto Encyclopedia of Genes and Genomes (KEGG) pathway enrichment analysis of differentially expressed genes (DEGs). The Benjamini–Hochberg adjusted p < 0.05 showed significant enrichment (20).

### 2.5 Immune cell analysis

To infer the composition of immune cells, the CIBERSORT algorithm was used to examine the relative proportions of 22 invasive immune cell types in each sample (21), and then the ggplot2 package was used for visualization.

### 2.6 Gene set variation analysis

According to previous studies, 23 genes, such as MPO, S100A8, and S100A9, were selected to constitute the functional characteristic gene set of neutrophil extracellular trap (NET) (22). Other functional signatures were derived from the GO database (see **Supplementary Table 1** for details). Finally, gene set variation analysis (GSVA) was performed using the GSVA package to obtain the score of each function, and the differences between groups were visualized using the ggplot2 package (20).

### 2.7 Weighted gene co-expression network analysis

Based on the gene expression profiling, the goodSamplesGenes method in the weighted gene co-expression network analysis (WGCNA) package was used to remove the outliers of genes and samples (23). Then, the median absolute deviation (MAD) of each gene was calculated to retain the top 50% of the genes. The Euclidean distance between the samples was calculated to remove an abnormal sample by clustering. β, a soft thresholding power that could emphasize strong correlations between genes and penalize weak correlations, was used. After choosing the best power, the adjacency was transformed into a topological overlap matrix (TOM), which could measure the network connectivity of a gene, defined as the sum of its adjacency with all other genes for the network gene ratio, and the corresponding dissimilarity (1 − TOM) was calculated. To classify genes with similar expression profiles into gene modules, average linkage hierarchical clustering was conducted according to the TOM-based dissimilarity measure, with a minimum size (gene group) of 100 for the gene dendrogram. To further analyze the module, the dissimilarity of module eigen genes was calculated, a cut line for the module dendrogram was chosen, and some modules were merged. The correlation coefficients and corresponding p-values between different modules and clinical traits were calculated and visualized using the heat map. The module showing the highest correlation with clinical features was identified as a key module along with the key genes. The top 10 hub genes were identified using the cytoHubba plug-in of Cytoscape (24).

### 2.8 Statistical analysis

All data were analyzed using R 4.4.0 and SPSS 26.0. Statistical descriptions were expressed as mean ± standard deviation (quantitative normal or near-normal data), median (P25, P75) (quantitative skewed data), and frequency (rate) (binary classification data). Two-group comparisons were analyzed for differences by t-test (quantitative normal or near-normal data), the Wilcoxon–Mann–Whitney rank-sum test (quantitative skewed data), and chi-square test (binary classification data) (p ≤ 0.05). Correlations were analyzed using Pearson’s or Spearman’s correlation coefficients (between quantitative data), coefficients of contingency (between binary classification data), and point biserial correlation coefficients (between binary classification and quantitative data) and then visualized using the corrplot package.

### 2.9 Ethics and informed consent

This study was approved by the Institutional Ethics Committee of Children’s Hospital of Nanjing Medical University and Qinghai University Affiliated Hospital (approval number: 202012089-1/P-SL-202111). All procedures performed in this study involving human participants were in accordance with the Declaration of Helsinki (as revised in 2013), and the informed consent of the parents or legal guardians of all enrolled children was obtained.

## 3 Results

### 3.1 Comparison of clinical characteristics among children with MPP at different altitudes

**Figure 1:**
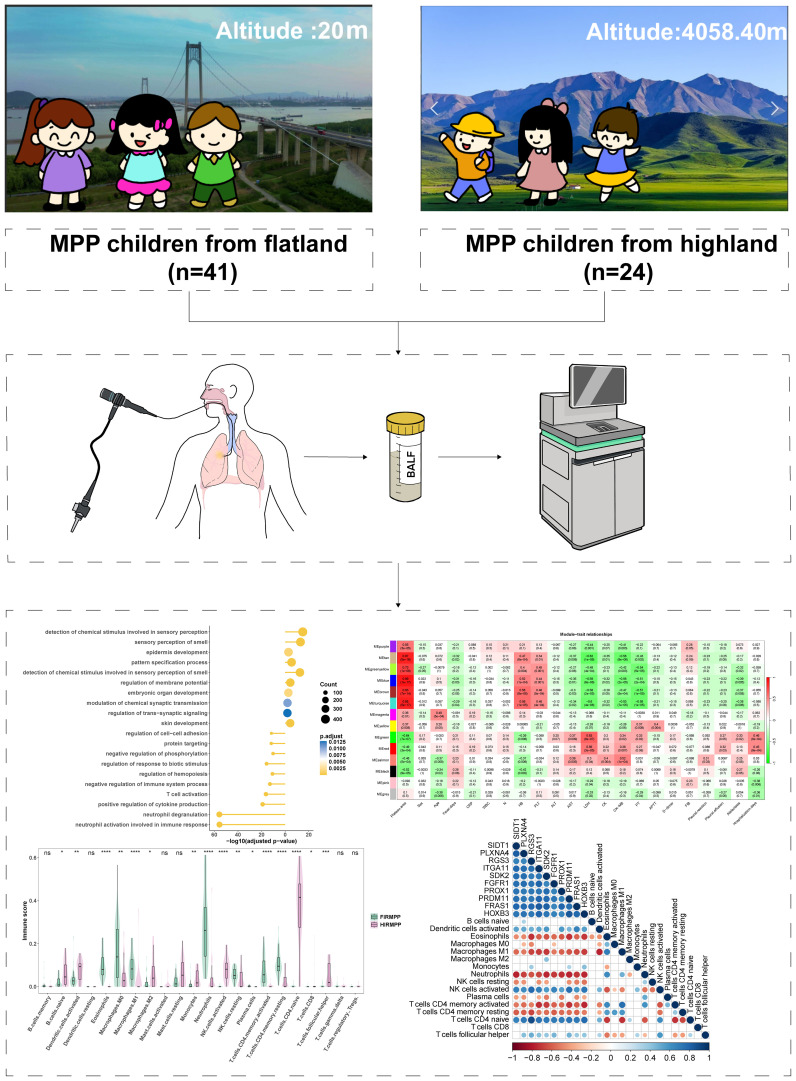
The overview of this study. MPP, *Mycoplasma pneumoniae* pneumonia; BALF, bronchoalveolar lavage fluid.

A total of 24 children with MPP from plateau areas [RMPP *vs*. general MPP (GMPP): 19:5] and 41 children with MPP from plain areas (RMPP *vs*. GMPP: 32:9) were enrolled in this study (**Figure 1**). Compared with the plateau group, children with RMPP in the plain group had longer duration of fever before admission; higher levels of aspartate aminotransferase (AST), lactate dehydrogenase (LDH), creatine kinase (CK), creatine kinase-MB (CK-MB), prothrombin time (PT), and CD4^+^ T cells; and increased incidence of atelectasis, but lower levels of hemoglobin (HB), platelet (PLT), B cells, and CD8^+^ T cells. No significant differences were observed in other indicators (**Table 1**). Compared with the plateau group, children with GMPP in the plain group had higher activated partial thromboplastin time (APTT) and lower D-dimer (**Table 2**).

**Table 1:** Comparison of clinical characteristics among children with RMPP at different altitudes.

Clinical characteristics		FlRMPP (n = 32)	HlRMPP (n = 19)	p-Value
Age (years), mean (SD)		6.70±2.75	7.71±2.02	0.202
Sex	Male	19 (59.38%)	10 (52.63%)	0.638
Duration of fever (days), mean (SD)		7.81±3.43	5.88±2.78	0.016*
Hospitalization days (days), mean (SD)		12.16±4.64	12.24±2.93	0.859
CRP (mg/L), median (P25, P75)		13.00 (2.83, 32.00)	6.07 (2.57, 37.60)	0.557
WBC (10^9^/L), mean (SD)		9.80±4.08	10.34±5.78	0.875
N (10^9^/L), median (P25, P75)		5.82 (4.32, 8.07)	4.71 (3.55, 9.94)	0.36
HB (g/L), mean (SD)		124.16±9.65	136.65±11.10	<0.001*
PLT (10^9^/L), mean (SD)		279.48±78.25	357.94±113.53	0.006*
ALT (U/L), median (P25, P75)		18.00 (14.00, 30.00)	20.00 (16.50, 40.50)	0.541
AST (U/L), mean (SD)		38.16±21.52	24.29±5.45	0.002*
LDH (U/L), mean (SD)		465.45±173.63	248.18±48.88	<0.001*
CK (U/L), median (P25, P75)		73.00 (34.00, 169.00)	31.00 (26.00, 52.00)	0.002*
CK-MB (U/L), mean (SD)		22.39±6.62	14.29±4.55	<0.001*
PT (s), mean (SD)		13.01±1.23	11.12±3.07	0.005*
APTT (s), mean (SD)		29.85±4.65	28.61±6.21	0.507
D-dimer (ng/mL), median (P25, P75)		812.00 (396.00, 2,015.00)	800.00 (600.00, 1,800.00)	0.592
Fbg (g/L), mean (SD)		3.64±0.61	3.69±1.10	0.783
T cell%, mean (SD)		66.43±8.82	60.92±8.07	0.303
CD4^+^ T cell%, mean (SD)		35.28±7.31	26.29±5.62	0.012*
CD8^+^ T cell%, mean (SD)		28.89±8.58	29.61±6.77	0.787
NK cell%, mean (SD)		12.60±6.65	11.33±4.78	0.233
B cell%, mean (SD)		17.21±8.44	25.94±7.98	0.010*
CD4^+^/CD8^+^ T cell, mean (SD)		1.36±0.60	0.94±0.32	0.084
T cell (/μL), mean (SD)		1,133.48±602.46	1,309.68±522.78	0.394
CD4^+^ T cell (/μL), mean (SD)		598.87±377.03	570.50±250.52	0.816
CD8^+^ T cell (/μL), mean (SD)		453.68±228.46	632.98±266.56	0.043*
NK cell (/μL), median (P25, P75)		180.97 (86.78, 387.63)	194.45 (150.40, 266.83)	0.383
B cell (/μL), mean (SD)		292.26±227.46	562.67±290.46	0.004*
Pleural reaction	Yes	5 (15.63%)	0	0.184
Pleural effusion	Yes	12 (37.50%)	3 (15.79%)	0.1
Atelectasis	Yes	11 (34.38%)	1 (5.26%)	0.043*

RMPP, refractory *Mycoplasma pneumoniae* pneumonia; FlRMPP, RMPP from flatlands; HlRMPP, RMPP from highlands; CRP, C-reactive protein; WBC, white blood cell; N, neutrophil; HB, hemoglobin; PLT, platelet; ALT, alanine aminotransferase; AST, aspartate aminotransferase; LDH, lactate dehydrogenase; CK, creatine kinase; CK-MB, creatine kinase-MB; PT, prothrombin time; APTT, activated partial thromboplastin time; Fbg, fibrinogen; NK cell, natural killer cell.^*^ p ≤ 0.05.

**Table 2:** Comparison of clinical characteristics among children with GMPP at different altitudes.

Clinical characteristics		FlGMPP (n = 9)	HlGMPP (n = 5)	p-Value
Age (years), mean (SD)		8.06±3.07	11.00±2.94	0.237
Sex	Male	6 (66.67%)	2 (40.00%)	0.58
Duration of fever (days), mean (SD)		8.67±2.45	5.25±3.95	0.102
Hospitalization days (days), mean (SD)		8.67±2.45	10.75±2.87	0.304
CRP (mg/L), mean (SD)		9.02±10.75	8.20±9.40	0.754
WBC (10^9^/L), mean (SD)		8.71±3.76	7.85±1.21	0.81
N (10^9^/L), mean (SD)		5.91±3.66	5.64±1.58	0.97
HB (g/L), mean (SD)		130.56±15.00	147.50±16.46	0.056
PLT (10^9^/L), mean (SD)		314.00±193.51	297.00±118.01	0.889
ALT (U/L), mean (SD)		13.00±6.27	29.75±19.43	0.055
AST (U/L), mean (SD)		31.00±11.91	24.00±8.17	0.233
LDH (U/L), mean (SD)		422.56±265.09	256.00±56.83	0.199
CK (U/L), mean (SD)		125.11±66.07	68.00±65.09	0.089
CK-MB (U/L), mean (SD)		30.44±40.91	8.00±3.16	0.311
PT (s), mean (SD)		12.26±1.17	10.88±1.19	0.077
APTT (s), mean (SD)		33.77±4.33	27.83±1.83	0.025*
D-dimer (ng/mL), median (P25, P75)		150.00 (119.50, 380.00)	2,360.00 (805.00, 2,475.00)	0.045*
Fbg (g/L), mean (SD)		3.50±0.63	3.68±0.99	0.694
Pleural effusion	Yes	1 (11.11%)	1 (20.00%)	1

GMPP, general *Mycoplasma pneumoniae* pneumonia; FlGMPP, GMPP from flatlands; HlGMPP, GMPP from highlands; CRP, C-reactive protein; WBC, white blood cell; N, neutrophil; HB, hemoglobin; PLT, platelet; ALT, alanine aminotransferase; AST, aspartate aminotransferase; LDH, lactate dehydrogenase; CK, creatine kinase; CK-MB, creatine kinase-MB; PT, prothrombin time; APTT, activated partial thromboplastin time; Fbg, fibrinogen; NK cell, natural killer cell.^*^ p ≤ 0.05.

### 3.2 Pulmonary immune responses of children with MPP in the plain region were excessively activated

**Figure 2:**
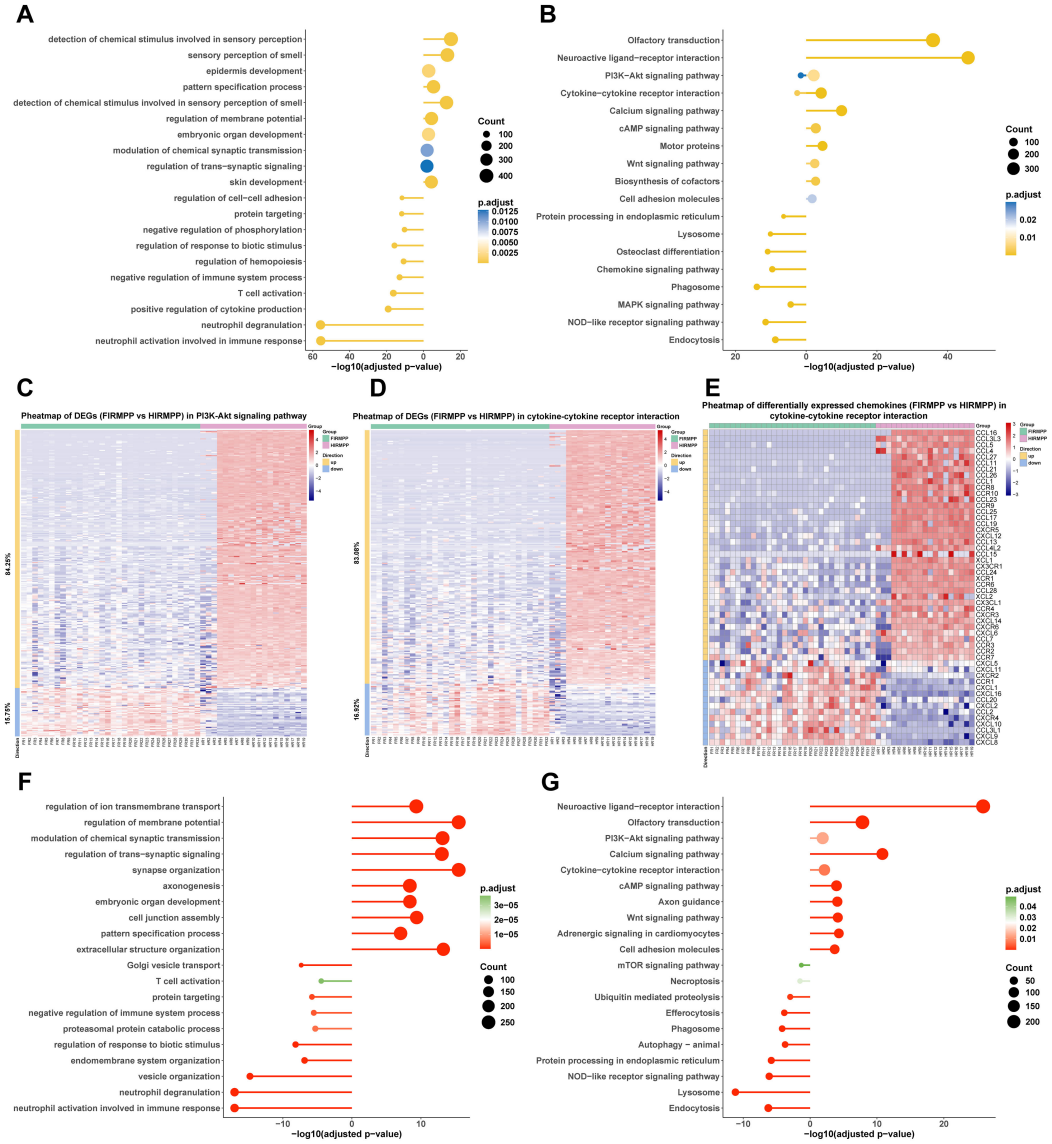
Pulmonary immune responses of children with MPP in the plain region were excessively activated. **(A)** GO_BP dot plot of DEGs in BALF from children with RMPP between the plateau and plain groups. The horizontal line moving to the right represents the upregulated DEGs, and the horizontal line moving to the left represents the downregulated DEGs. The size of the circle represents the number of enriched genes. Benjamini–Hochberg adjusted p-value < 0.05 shows significant enrichment. **(B)** KEGG dot plot of DEGs in BALF from children with RMPP between the plateau and plain groups. **(C)** Heat map of DEGs in PI3K–Akt signaling pathway in BALF from children with RMPP between the plateau and plain groups. The redder the color, the higher the DEG expression. **(D)** Heat map of DEGs in cytokine–cytokine receptor interaction in BALF from children with RMPP between the plateau and plain groups. **(E)** Heat map of differentially expressed chemokines in cytokine–cytokine receptor interaction in BALF from children with RMPP between the plateau and plain groups. **(F)** GO_BP dot plot of DEGs in BALF from children with GMPP between the plateau and plain groups. **(G)** KEGG dot plot of DEGs in BALF from children with GMPP between the plateau and plain groups. MPP, *Mycoplasma pneumoniae* pneumonia; GO, Gene Ontology; BP, biological process; DEGs, differentially expressed genes; BALF, bronchoalveolar lavage fluid; RMPP, refractory *M. pneumoniae* pneumonia; KEGG, Kyoto Encyclopedia of Genes and Genomes; GMPP, general *M. pneumoniae* pneumonia; FlRMPP, RMPP from flatlands; HlRMPP, RMPP from highlands.

To better understand the differences in the host transcriptional levels of children with MPP in the plateau and plain regions, we compared the levels of gene expression in BALF from children with MPP between the plateau and plain groups. A total of 23,566 DEGs were identified in BALF from children with RMPP between the plateau and plain groups, with 21,654 upregulated genes (**Supplementary Figures 1A, B**). GO enrichment analysis revealed that downregulated DEGs in the plateau group were significantly enriched in neutrophil activation involved in immune response, neutrophil degranulation, and T-cell activation (**Figure 2A**). KEGG enrichment analysis showed that upregulated DEGs in the plateau group were significantly enriched in the PI3K–Akt signaling pathway, cytokine–cytokine receptor interaction, and calcium signaling pathway, while downregulated DEGs were significantly enriched in endocytosis, the NOD-like receptor signaling pathway, the MAPK signaling pathway, and the chemokine signaling pathway. Both upregulated and downregulated DEGs in the plateau group were significantly enriched in the PI3K–Akt signaling pathway and cytokine–cytokine receptor interaction (**Figure 2B**). Among DEGs significantly enriched in the PI3K–Akt signaling pathway and cytokine–cytokine receptor interaction, the number of upregulated DEGs in the plateau group was significantly higher (**Figures 2C, D**). In addition, among DEGs significantly enriched in the cytokine–cytokine receptor interaction, CXCL1, CXCL2, CXCL8, and CXCR2 were significantly downregulated in the plateau group, while XCL1, XCL2, and XCR1 were significantly upregulated in the plateau group (**Figure 2E**).

Meanwhile, a total of 12,903 DEGs were identified in BALF from children with GMPP between the plateau and plain groups, with 11,389 upregulated genes (**Supplementary Figures 1C, D**). GO enrichment analysis revealed that downregulated DEGs in the plateau group were also significantly enriched in neutrophil activation involved in immune response, neutrophil degranulation, and T-cell activation (**Figure 2F**). KEGG enrichment analysis found that the upregulated DEGs in the plateau group were significantly enriched in the PI3K–Akt signaling pathway, the calcium signaling pathway, and cytokine–cytokine receptor interaction, while downregulated DEGs were significantly enriched in endocytosis, the NOD-like receptor signaling pathway, and efferocytosis (**Figure 2G**).

### 3.3 Children with RMPP in the plateau region exhibited increased M2 macrophages and CD8^+^ T cells, and decreased M1 macrophages, neutrophils, and CD4^+^ T cells

**Figure 3:**
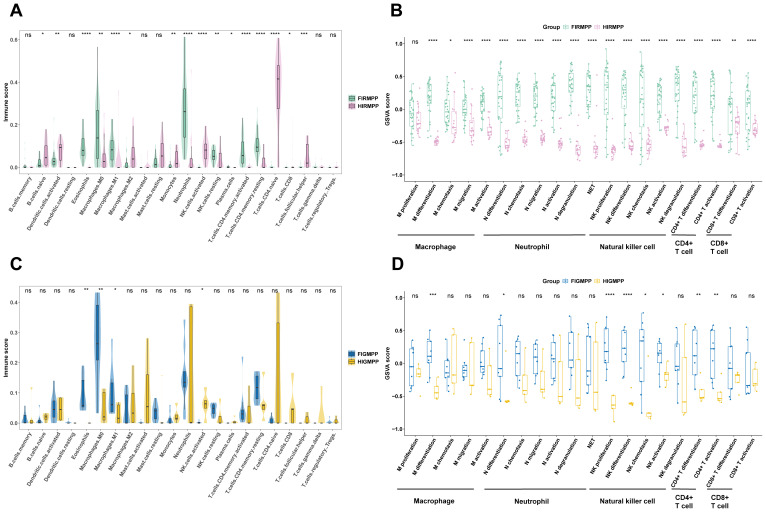
Children with RMPP in the plateau region exhibited increased M2 macrophages and CD8+ T cells, and decreased M1 macrophages, neutrophils and CD4+ T cells. **(A)** Proportion of pulmonary immune cells in BALF from children with RMPP between the plateau and plain groups based on transcriptome data. **(B)** The functional gene set scores of immune cells in BALF from children with RMPP between the plateau and plain groups. **(C)** Proportion of pulmonary immune cells in BALF from children with GMPP between the plateau and plain groups based on transcriptome data. **(D)** The functional gene set scores of immune cells in BALF from children with GMPP between the plateau and plain groups. RMPP, refractory Mycoplasma pneumoniae pneumonia; BALF, bronchoalveolar lavage fluid; FlRMPP, RMPP from flatlands; HlRMPP, RMPP from highlands; FlGMPP, GMPP from flatlands; HlGMPP, GMPP from highlands; ****, P ≤ 0.0001; ***, P ≤ 0.001; **, P ≤ 0.01; *, P ≤ 0.05; ns, P > 0.05.

To further investigate whether there were differences in the pulmonary immune cells of children with MPP at different altitudes, CIBERSORT was used to reveal that the levels of naive B cells, activated dendritic cells, M2 macrophages, monocytes, activated NK cells, naive CD4^+^ T cells, CD8^+^ T cells, and follicular helper T cells in BALF from children with RMPP in the plateau group were significantly increased. However, the levels of eosinophils, M0 and M1 macrophages, neutrophils, resting NK cells, and memory CD4^+^ T cells in BALF from children with RMPP in the plateau group were significantly decreased (**Figure 3A**). The functional gene set scores of macrophages, neutrophils, NK cells, CD4^+^ T cells, and CD8^+^ T cells were significantly decreased in children with RMPP in the plateau group (**Figure 3B**). In addition, children with GMPP in the plateau group had higher levels of activated NK cells, but lower levels of eosinophils, and M0 and M1 macrophages in BALF (**Figure 3C**). The functional gene set scores for the differentiation of macrophages and neutrophils, NK cells, and CD4^+^ T cells in children with GMPP in the plateau group were significantly decreased (**Figure 3D**).

### 3.4 The association between key genes most closely related to the high altitude and pulmonary immune cells of children with MPP

**Figure 4:**
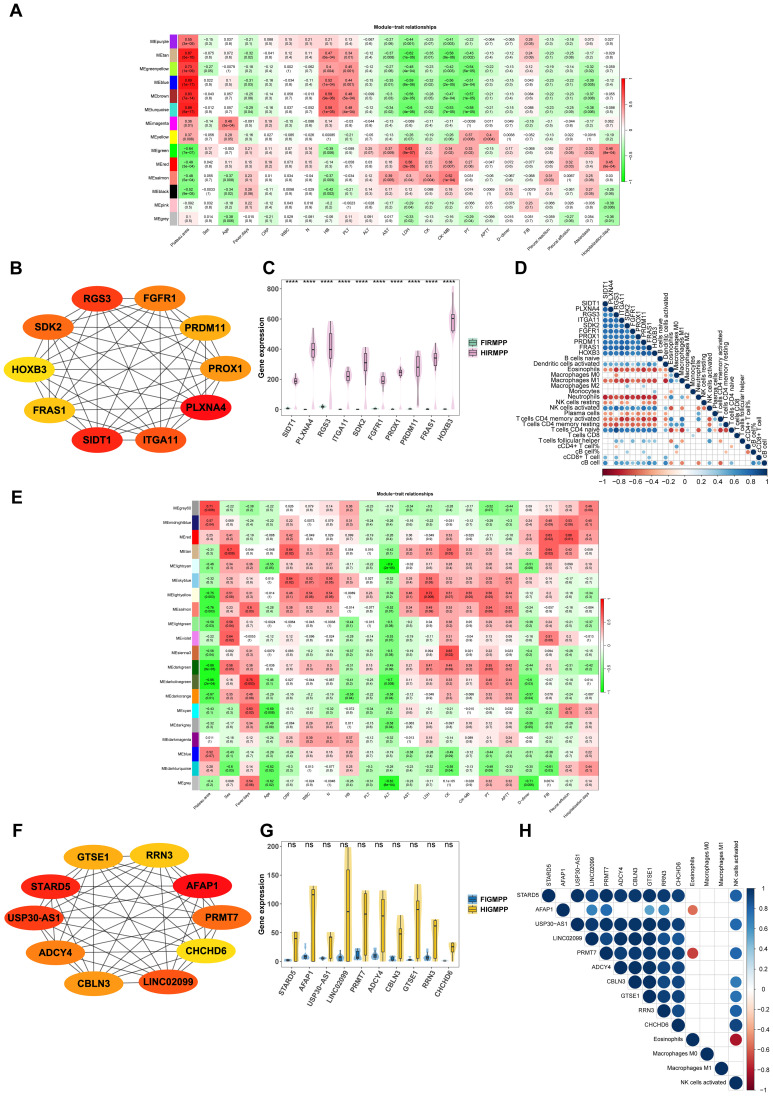
The association between key genes most closely related to the high altitude and pulmonary immune cells in children with MPP. **(A)** Heat map of correlation coefficients and corresponding P-values between different modules and clinical traits in children with RMPP. **(B)** Top 10 hub genes of the blue module identified by using the cytoHubba plug-in of Cytoscape. **(C)** Comparison of top 10 hub genes in the blue module between the plateau and plain groups. **(D)** Correlation analysis of top 10 hub genes in the blue module and pulmonary immune cells of children with RMPP.ÐE)Heat map of correlation coefficients and corresponding P-values between different modules and clinical traits in children with GMPP. **(F)** Top 10 hub genes of the grey60 module identified by using the cytoHubba plug-in of Cytoscape. **(G)** Comparison of top 10 hub genes in the grey60 module between the plateau and plain groups. **(H)** Correlation analysis of top 10 hub genes in the grey60 module and pulmonary immune cells of children with GMPP.ÐPP, Mycoplasma pneumoniae pneumonia; RMPP, refractory Mycoplasma pneumoniae pneumonia; GMPP, general Mycoplasma pneumoniae pneumonia; ****, P ≤ 0.0001; ns, P > 0.05.

To further explore the association between the levels of gene expression and different altitudes, WGCNA was used to identify that the gene expression in the blue module was most closely associated with whether children with RMPP were from the plateau regions (**Figure 4A**). Ten key genes (hub genes) of the blue module were identified using cytoHubba in Cytoscape, including SIDT1, PLXNA4, RGS3, ITGA11, SDK2, FGFR1, PROX1, PRDM11, FRAS1, and HOXB3 (**Figure 4B**). The expression of these 10 hub genes was all significantly upregulated in the plateau group (**Figure 4C**). The levels of eosinophils, M1 macrophages, neutrophils, and memory CD4^+^ T cells showed significant negative correlations with the 10 hub genes, while the levels of activated NK cells and naive CD4^+^ T cells exhibited significant positive correlations with the 10 hub genes (**Figure 4D**). In addition, the gene expression of the grey60 module was most closely associated with whether children with GMPP were from the plateau regions (**Figure 4E**). Ten key genes (hub genes) of the grey60 module were identified using cytoHubba in Cytoscape, including STARD5, AFAP1, USP30-AS1, LINC02099, PRMT7, ADCY4, CBLN3, GTSE1, RRN3, and CHCHD6 (**Figure 4F**). The expression of these 10 hub genes was higher in the plateau group, but the differences were not statistically significant (**Figure 4G**). The levels of AFAP1 and PRMT7 exhibited significant negative correlations with eosinophils, while activated NK cells showed significant positive correlations with all hub genes except AFAP1, LINC02099, and ADCY4 (**Figure 4H**).

### 3.5 The intersection of DEGs and genes in the WGCNA module most relevant to the high altitude was used to identify the characteristic pathways of children with MPP in the plateau regions

**Figure 5:**
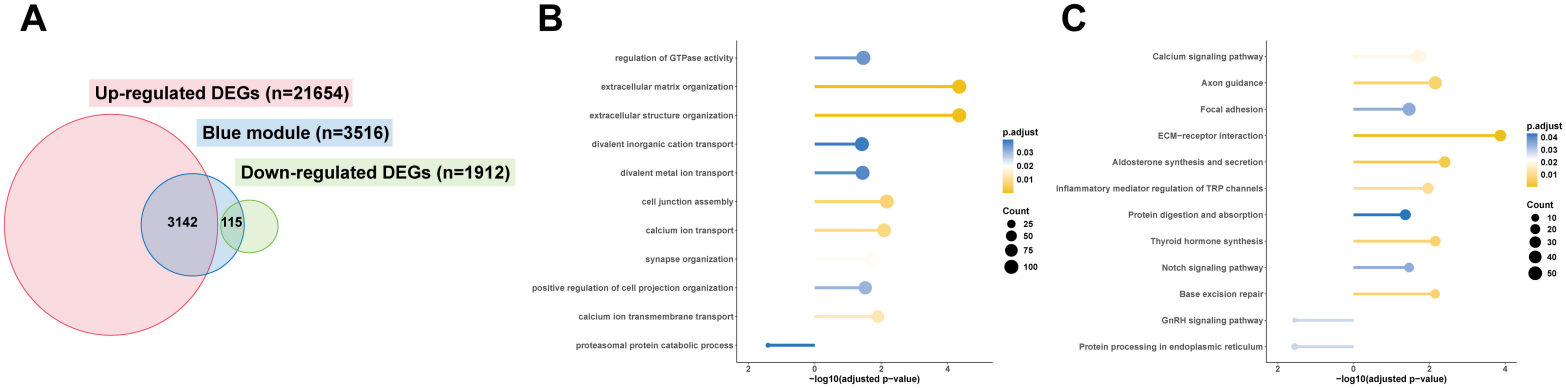
The intersection of DEGs and genes in the WGCNA module most relevant to the high altitude was used to identify the characteristic pathways of children with RMPP in the plateau regions. **(A)** The intersection of DEGs and genes in the WGCNA module most relevant to the high altitude. **(B)** GO_BP dot plot of DEGs most related to the plateau regions in the RMPP group. The horizontal line moving to the right represents the up-regulated DEGs, and the horizontal line moving to the left represents the down-regulated DEGs. The size of the circle represents the number of enriched genes. Benjamini-Hochberg adjusted p value < 0.05 shows significant enrichment. **(C)** KEGG dot plot of DEGs most related to the plateau regions in the RMPP group. DEGs, differentially expressed genes; WGCNA, Weighted gene co-expression network analysis; RMPP, refractory Mycoplasma pneumoniae pneumonia; KEGG, Kyoto Encyclopedia of Genes and Genomes.

The intersection of DEGs and genes in the blue module of children with RMPP between the plateau and plain groups yielded a total of 3,142 upregulated and 115 downregulated DEGs most related to the plateau regions (**Figure 5A**). GO enrichment analysis revealed that the upregulated DEGs most related to the plateau regions in the RMPP group were significantly enriched in the regulation of GTPase activity, extracellular matrix organization, and calcium ion transport (**Figure 5B**). KEGG enrichment analysis found that the upregulated DEGs most associated with the plateau regions in the RMPP group were significantly enriched in the calcium signaling pathway, axon guidance, and extracellular matrix (ECM)–receptor interaction (**Figure 5C**). Similarly, the intersection of DEGs and genes in the grey60 module of children with GMPP between the plateau and plain groups yielded 177 upregulated DEGs most related to the plateau regions, but pathway enrichment analysis did not detect significant enrichment in specific pathways (**Supplementary Figure 2**).

## 4 Discussion

MPP is one of the most common respiratory infectious diseases in children. In 2023, a rare global outbreak of MPP occurred, accompanied by a large number of poor prognoses such as RMPP, pulmonary embolism, and necrotizing pneumonia (4–7). Previous studies have shown that children in high-altitude regions have a higher incidence of pneumonia, but the severity of pneumonia is lower (16–18). This study also found that children with RMPP in plateau areas had milder clinical manifestations, such as shorter duration of fever before admission, lower levels of serum tissue injury markers, and lower incidences of atelectasis. In addition, high-altitude climate therapy can significantly alleviate clinical manifestations in children with allergic asthma (15). Therefore, children who have lived in high-altitude regions for a long time may experience adaptive alterations in immune functions due to unique environmental factors.

This study performed transcriptome sequencing of BALF from children with MPP in the plateau and plain regions. Significantly downregulated activation of neutrophils, T cells, and the NOD-like receptor signaling pathways, and upregulated activation of the PI3K–Akt signaling pathway were observed in the lungs of children with MPP in plateau areas. Transcriptome studies of BALF from children with MPP have shown excessive activation and proliferation of T cells as well as the upregulation of related genes (25, 26). Our self-controlled model of MPP demonstrated that the side with severe lung lesions had more infiltrated neutrophils and more significant activation of neutrophils. Then, the upregulated DEGs in the MPP group were significantly enriched in the activation of neutrophils and the NOD-like receptor signaling pathway, and the activation of neutrophils was more significant in the severe MPP group by transcriptome sequencing of peripheral neutrophils from healthy children and those with severe or mild MPP (20). NOD-like receptors are the main intracellular pattern recognition receptors, which are key immune receptors in innate immune responses (27). NOD2 is significantly expressed in neutrophils, which can recruit neutrophils and macrophages to the sites of infections and cause tissue damage by inducing chemokines (28, 29). The PI3K–Akt signaling pathway is mainly associated with the polarization of macrophages and immune suppression in pulmonary diseases. The M2 polarization of macrophages observed in patients with chronic obstructive pulmonary disease (COPD) is mediated by the activation of the PTEN/PI3K/AKT pathway (30). Carbon dots (CDots) can enhance phagocytosis by inhibiting the PI3K signaling pathway and promoting M1 polarization of macrophages during methicillin-resistant *Staphylococcus aureus* (MRSA) or *Klebsiella pneumoniae* infections (31). The severe acute respiratory syndrome coronavirus-2 (SARS-CoV-2)S protein inhibits inflammatory responses of alveolar epithelial type II cells during the early stages of infections by activating the PI3K/AKT pathway (32). The SARS-CoV-2 spike induces autophagy by upregulating intracellular reactive oxygen species (ROS) and subsequently inhibiting the PI3K/AKT/mTOR axis, leading to inflammatory responses and apoptosis in infected cells (33). We speculate that the high-altitude exposure primarily suppresses pulmonary immune functions in children with MPP, manifested as downregulated activation of neutrophils and T cells, which may be associated with the inhibition of the NOD-like receptor signaling pathway or the activation of the PI3K–Akt signaling pathway.

Previous studies have shown that children with MPP have increased levels of circulating NK cells, activated regulatory T cells, functional B cells, and neutrophils, but reduced levels of CD8^+^ T cells and immature B cells (34). CD4^+^ T cells and neutrophils in BALF from children with MPP increase significantly, while CD8^+^ T cells decrease greatly (26). B-cell functional pathways in BALF from children with severe MPP are downregulated (35). Residents in high-altitude regions exhibit reduced levels of circulating B cells, CD4^+^ T cells, and dendritic cells, but increased levels of CD8^+^ T cells and NK cells (8–10). Our study demonstrated that circulating CD4^+^ T cells in children with RMPP of the plateau group were decreased, while CD8^+^ T cells and B cells were significantly increased. In addition, M0 and M1 macrophages, neutrophils, and memory CD4^+^ T cells in the lungs of children with RMPP in the plateau group were significantly decreased, while activated dendritic cells, M2 macrophages, activated NK cells, CD8^+^ T cells, and immature B cells were significantly increased. Thus, adaptive changes may appear in immune cells of children with MPP in high-altitude areas, manifested as increased M2 polarization of macrophages, CD8^+^ T cells, and B cells, and decreased neutrophils and CD4^+^ T cells.

This study has some limitations. First, the sample size was relatively small, and the enrollment times were inconsistent between the two groups. This was because fiberoptic bronchoscopy is an invasive procedure, and it was difficult to collect BALF from children with MPP. The sample size can be expanded for further analysis in the future. Second, flow cytometry or single-cell sequencing may be adopted to further verify the pulmonary immune cells of children with MPP at different altitudes. In addition, transcriptome analysis demonstrated the impact of the altitude on the immunological characteristics and possible mechanisms of children with MPP. Corresponding experiments can be carried out in the future for verification.

## 5 Conclusion

Excessive activation of the pulmonary immune response in children with MPP of the plain region, characterized by the activation of neutrophils and T cells, may be related to the NOD-like receptor signaling pathway and PI3K–Akt signaling pathway. Adaptive changes occurred in immune cells of children with MPP in the plateau region, manifested as increased M2 polarization of macrophages, CD8^+^ T cells, and B cells, and decreased neutrophils and CD4^+^ T cells.

## Data Availability

The datasets presented in this study can be found in online repositories. The names of the repository/repositories and accession number(s) can be found in the article/**Supplementary Material**.

## References

[1] Otheo E Rodríguez M Moraleda C Domínguez-Rodríguez S Martín MD Herreros ML . Viruses and Mycoplasma pneumoniae are the main etiological agents of community-acquired pneumonia in hospitalized pediatric patients in Spain. Pediatr Pulmonol. (2022) 57:253–63. doi: 10.1002/ppul.25721, PMID: 34633153

[2] Cheng Y Cheng Y Dai S Hou D Ge M Zhang Y . The prevalence of mycoplasma pneumoniae among children in beijing before and during the COVID-19 pandemic. Front Cell Infect Microbiol. (2022) 12:854505. doi: 10.3389/fcimb.2022.854505, PMID: 35573799 PMC9103471

[3] National Health Commission of the People’s Republic of China . Guidelines for the diagnosis and treatment of Mycoplasma pneumoniae pneumonia in children (2023 edition). Int J Epidemiol. (2023) 50:79–85. doi: 10.3760/cma.j.cn331340-20230217-00023

[4] Diaz MH Hersh AL Olson J Shah SS Hall M Edens C . Mycoplasma pneumoniae infections in hospitalized children - United States, 2018-2024. MMWR Morb Mortal Wkly Rep. (2025) 74:394–400. doi: 10.15585/mmwr.mm7423a1, PMID: 40569891 PMC12200605

[5] Wang W Wang L Guo R Bo Y Zou Y Cui H . Epidemiological association of the COVID-19 pandemic on Mycoplasma pneumoniae infections in children in Tianjin, China: a single-centre retrospective study (2017-2024). BMJ Open. (2025) 15:e101045. doi: 10.1136/bmjopen-2025-101045, PMID: 40527576 PMC12182171

[6] Qi J Li H Li H Wang Z Zhang S Meng X . Epidemiological changes of Mycoplasma pneumoniae among children before, during, and post the COVID-19 pandemic in Henan, China, from 2017 to 2024. Microbiol Spectr. (2025) 13:e0312124. doi: 10.1128/spectrum.03121-24, PMID: 40488359 PMC12252384

[7] Mao J Niu Z Liu M Li L Zhang H Li R . Comparison of the epidemiological characteristics of mycoplasma pneumoniae infections among children during two epidemics in Wuhan from 2018 to 2024. BMC Pediatr. (2025) 25:71. doi: 10.1186/s12887-025-05435-9, PMID: 39875866 PMC11773782

[8] Bai J Li L Li Y Zhang L . Genetic and immune changes in Tibetan high-altitude populations contribute to biological adaptation to hypoxia. Environ Health Prev Med. (2022) 27:39. doi: 10.1265/ehpm.22-00040, PMID: 36244759 PMC9640738

[9] Rohm I Aderhold N Ratka J Goebel B Franz M Pistulli R . Hypobaric hypoxia in 3000 m altitude leads to a significant decrease in circulating plasmacytoid dendritic cells in humans. Clin Hemorheol Microcirc. (2016) 63:257–65. doi: 10.3233/CH-152035, PMID: 26890107

[10] Facco M Zilli C Siviero M Ermolao A Travain G Baesso I . Modulation of immune response by the acute and chronic exposure to high altitude. Med Sci Sports Exerc. (2005) 37:768–74. doi: 10.1249/01.mss.0000162688.54089.ce, PMID: 15870630

[11] Zhang X Li J Hu F Gao S Pu X Yong S . Decreased number and immune activity of splenic T lymphocytes in mice exposed to hypoxia at high altitude. Xi Bao Yu Fen Zi Mian Yi Xue Za Zhi. (2017) 33:164–7. doi: 10.13423/j.cnki.cjcmi.008007, PMID: 29762003

[12] Tiollier E Schmitt L Burnat P Fouillot JP Robach P Filaire E . Living high-training low altitude training: effects on mucosal immunity. Eur J Appl Physiol. (2005) 94:298–304. doi: 10.1007/s00421-005-1317-4, PMID: 15765238

[13] Karagiannidis C Hense G Rueckert B Mantel PY Ichters B Blaser K . High-altitude climate therapy reduces local airway inflammation and modulates lymphocyte activation. Scand J Immunol. (2006) 63:304–10. doi: 10.1111/j.1365-3083.2006.01739.x, PMID: 16623931

[14] Boonpiyathad T Capova G Duchna HW Croxford AL Farine H Dreher A . Impact of high-altitude therapy on type-2 immune responses in asthma patients. Allergy. (2020) 75:84–94. doi: 10.1111/all.13967, PMID: 31267528

[15] Simon HU Grotzer M Nikolaizik WH Blaser K Schöni MH . High altitude climate therapy reduces peripheral blood T lymphocyte activation, eosinophilia, and bronchial obstruction in children with house-dust mite allergic asthma. Pediatr Pulmonol. (1994) 17:304–11. doi: 10.1002/ppul.1950170507, PMID: 8058424

[16] Ortiz-Prado E Cortez-Silva MV Vasconez-Gonzalez J Izquierdo-Condoy JS Peñafiel J Crookston BT . Pediatric pneumonia across altitudes in Ecuador: a countrywide, epidemiological analysis from 2010-2021. Ital J Pediatr. (2025) 51:165. doi: 10.1186/s13052-025-02004-9, PMID: 40442808 PMC12123815

[17] Khan AJ Hussain H Omer SB Chaudry S Ali S Khan A . High incidence of childhood pneumonia at high altitudes in Pakistan: a longitudinal cohort study. Bull World Health Organ. (2009) 87:193–9. doi: 10.2471/blt.07.048264, PMID: 19377715 PMC2654635

[18] Simbaña-Rivera K Jaramillo PRM Silva JVV Gómez-Barreno L Campoverde ABV Novillo Cevallos JF . High-altitude is associated with better short-term survival in critically ill COVID-19 patients admitted to the ICU. PloS One. (2022) 17:e0262423. doi: 10.1371/journal.pone.0262423, PMID: 35358185 PMC8970356

[19] National Health Commission of the People’s Republic of China State Administration of Traditional Chinese Medicine . Guideline for diagnosis and treatment of community-acquired pneumonia in Children (2019 version). Chin J Clin Infect Dis. (2019) 12:6–13. doi: 10.3760/cma.j.issn.1674-2397.2019.01.002

[20] Zhu Y Luo Y Li L Jiang X Du Y Wang J . Immune response plays a role in Mycoplasma pneumoniae pneumonia. Front Immunol. (2023) 14:1189647. doi: 10.3389/fimmu.2023.1189647, PMID: 37304280 PMC10250694

[21] Newman AM Liu CL Green MR Gentles AJ Feng W Xu Y . Robust enumeration of cell subsets from tissue expression profiles. Nat Methods. (2015) 12:453–7. doi: 10.1038/nmeth.3337, PMID: 25822800 PMC4739640

[22] Shen XT Xie SZ Xu J Yang LY Qin LX . Pan-cancer analysis reveals a distinct neutrophil extracellular trap-associated regulatory pattern. Front Immunol. (2022) 13:798022. doi: 10.3389/fimmu.2022.798022, PMID: 35432310 PMC9009150

[23] Langfelder P Horvath S . WGCNA: an R package for weighted correlation network analysis. BMC Bioinf. (2008) 9:559. doi: 10.1186/1471-2105-9-559, PMID: 19114008 PMC2631488

[24] Shannon P Markiel A Ozier O Baliga NS Wang JT Ramage D . Cytoscape: a software environment for integrated models of biomolecular interaction networks. Genome Res. (2003) 13:2498–504. doi: 10.1101/gr.1239303, PMID: 14597658 PMC403769

[25] Gao M Wang K Yang M Meng F Lu R Zhuang H . Transcriptome analysis of bronchoalveolar lavage fluid from children with mycoplasma pneumoniae pneumonia reveals natural killer and T cell-proliferation responses. Front Immunol. (2018) 9:1403. doi: 10.3389/fimmu.2018.01403, PMID: 29967623 PMC6015898

[26] Chen X Liu F Zheng B Kang X Wang X Mou W . Exhausted and apoptotic BALF T cells in proinflammatory airway milieu at acute phase of severe mycoplasma pneumoniae pneumonia in children. Front Immunol. (2022) 12:760488. doi: 10.3389/fimmu.2021.760488, PMID: 35111152 PMC8801936

[27] Patel S . Inflammasomes, the cardinal pathology mediators are activated by pathogens, allergens and mutagens: A critical review with focus on NLRP3. BioMed Pharmacother. (2017) 92:819–25. doi: 10.1016/j.biopha.2017.05.126, PMID: 28599247

[28] Ekman AK Cardell LO . The expression and function of Nod-like receptors in neutrophils. Immunology. (2010) 130:55–63. doi: 10.1111/j.1365-2567.2009.03212.x, PMID: 20002790 PMC2855793

[29] Trindade BC Chen GY . NOD1 and NOD2 in inflammatory and infectious diseases. Immunol Rev. (2020) 297:139–61. doi: 10.1111/imr.12902, PMID: 32677123 PMC8928416

[30] Lu J Xie L Liu C Zhang Q Sun S . PTEN/PI3k/AKT regulates macrophage polarization in emphysematous mice. Scand J Immunol. (2017) 85:395–405. doi: 10.1111/sji.12545, PMID: 28273403

[31] Jiang X Wang J Gan L Wu Z Wu T Li F . Carbon dot-based treatment for bacterial pneumonia by promoting a PI3K-mediated M1 polarization of macrophages. J Nanobiotechnol. (2025) 23:315. doi: 10.1186/s12951-025-03399-7, PMID: 40287711 PMC12032645

[32] Al-Qahtani AA Pantazi I Alhamlan FS Alothaid H Matou-Nasri S Sourvinos G . SARS-CoV-2 modulates inflammatory responses of alveolar epithelial type II cells via PI3K/AKT pathway. Front Immunol. (2022) 13:1020624. doi: 10.3389/fimmu.2022.1020624, PMID: 36389723 PMC9659903

[33] Li F Li J Wang PH Yang N Huang J Ou J . SARS-CoV-2 spike promotes inflammation and apoptosis through autophagy by ROS-suppressed PI3K/AKT/mTOR signaling. Biochim Biophys Acta Mol Basis Dis. (2021) 1867:166260. doi: 10.1016/j.bbadis.2021.166260, PMID: 34461258 PMC8390448

[34] Jia R Guo H Lu A Zhang C Qi Y Wang D . Immunological landscape of children with Mycoplasma pneumoniae pneumonia in the post-COVID-19 era reveals distinctive severity indicators. Respir Res. (2025) 26:103. doi: 10.1186/s12931-025-03189-7, PMID: 40097989 PMC11917007

[35] Wang K Gao M Yang M Meng F Li D Lu R . Transcriptome analysis of bronchoalveolar lavage fluid from children with severe Mycoplasma pneumoniae pneumonia reveals novel gene expression and immunodeficiency. Hum Genomics. (2017) 11:4. doi: 10.1186/s40246-017-0101-y, PMID: 28302172 PMC5356355

