# Supplementary material for: High-altitude immune remodeling in children with Mycoplasma pneumoniae pneumonia: a multi-center transcriptome study
Source: Front Immunol. 2025 Dec 16;16:1704739. doi: 10.3389/fimmu.2025.1704739 (PMC12747907; doi:10.3389/fimmu.2025.1704739)
Supplement: Supplementary file 1 [file DataSheet1.docx]

Supplementary Table 1. **Functional gene set.**

| Gene set | Gene/GO number |
| --- | --- |
| macrophage proliferation | GO:0061517 |
| macrophage differentiation | GO:0030225 |
| macrophage chemotaxis | GO:0048246 |
| macrophage migration | GO:1905517 |
| macrophage activation | GO:0042116 |
| neutrophil differentiation | GO:0030223 |
| neutrophil chemotaxis | GO:0030593 |
| neutrophil migration | GO:1990266 |
| neutrophil activation | GO:0042119 |
| neutrophil degranulation | GO:0043312 |
| NET | ACTB, ACTG1, ACTN1, ACTN4, AZU1,  CAT, CTSG, DEFA3, ELANE, ENO1, KRT10,  LCP1, LTF, LYZ, MNDA, MPO, MYH9,  PADI4, PRTN3, S100A12, S100A8,  S100A9, TKT |
| natural killer cell proliferation | GO:0001787 |
| natural killer cell differentiation | GO:0001779 |
| natural killer cell chemotaxis | GO:0035747 |
| natural killer cell activation | GO:0030101 |
| natural killer cell degranulation | GO:0043320 |
| CD4^+^ T cell differentiation | GO:0043367 |
| CD4^+^ T cell activation | GO:0035710 |
| CD8^+^ T cell differentiation | GO:0043374 |
| CD8^+^ T cell activation | GO:0036037 |

GO, Gene Ontology; NET, neutrophil extracellular trap.


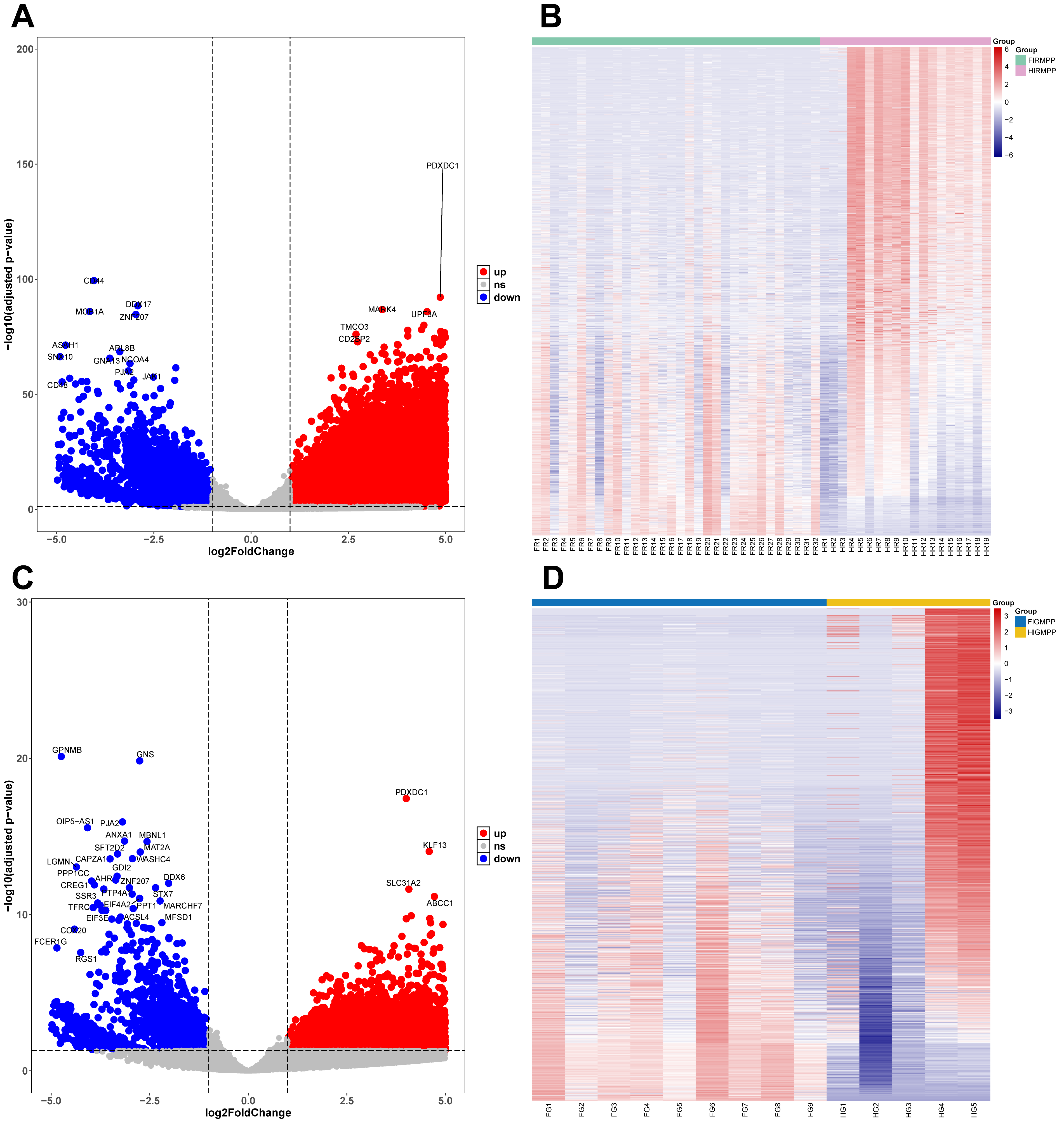


**Supplementary Figure S1. Differential expression analysis of BALF from children with MPP between the plateau and plain groups.**

1. Volcano map of DEGs in BALF from children with RMPP between the plateau and plain groups. Fold change was the ratio of the average expression of genes in the plateau group to those in the plain group, and an adjusted p value was obtained by the multiple hypothesis test, Benjamini-Hochberg method. The absolute value of fold change = 1 and the adjusted p value = 0.05 were used as cutoff values to determine the DEGs. When log2 (fold change) > 1 and adjusted p value < 0.05, DEGs were considered as up-regulated. The DEGs of log2 (fold change) < −1 and adjusted p value < 0.05 were considered down-regulated.
2. Heat map of DEGs in BALF from children with RMPP between the plateau and plain groups. The heat map was the log conversion of the DEG expression in each sample. The redder the color, the higher the DEG expression.
3. Volcano map of DEGs in BALF from children with GMPP between the plateau and plain groups.
4. Heat map of DEGs in BALF from children with GMPP between the plateau and plain groups.

MPP, *Mycoplasma pneumoniae* pneumonia; DEGs, differentially expressed genes; BALF, bronchoalveolar lavage fluid; RMPP, refractory *Mycoplasma pneumoniae* pneumonia; GMPP, general *Mycoplasma pneumoniae* pneumonia; FlRMPP, RMPP from flatlands; HlRMPP, RMPP from highlands; FlGMPP, GMPP from flatlands; HlGMPP, GMPP from highlands.


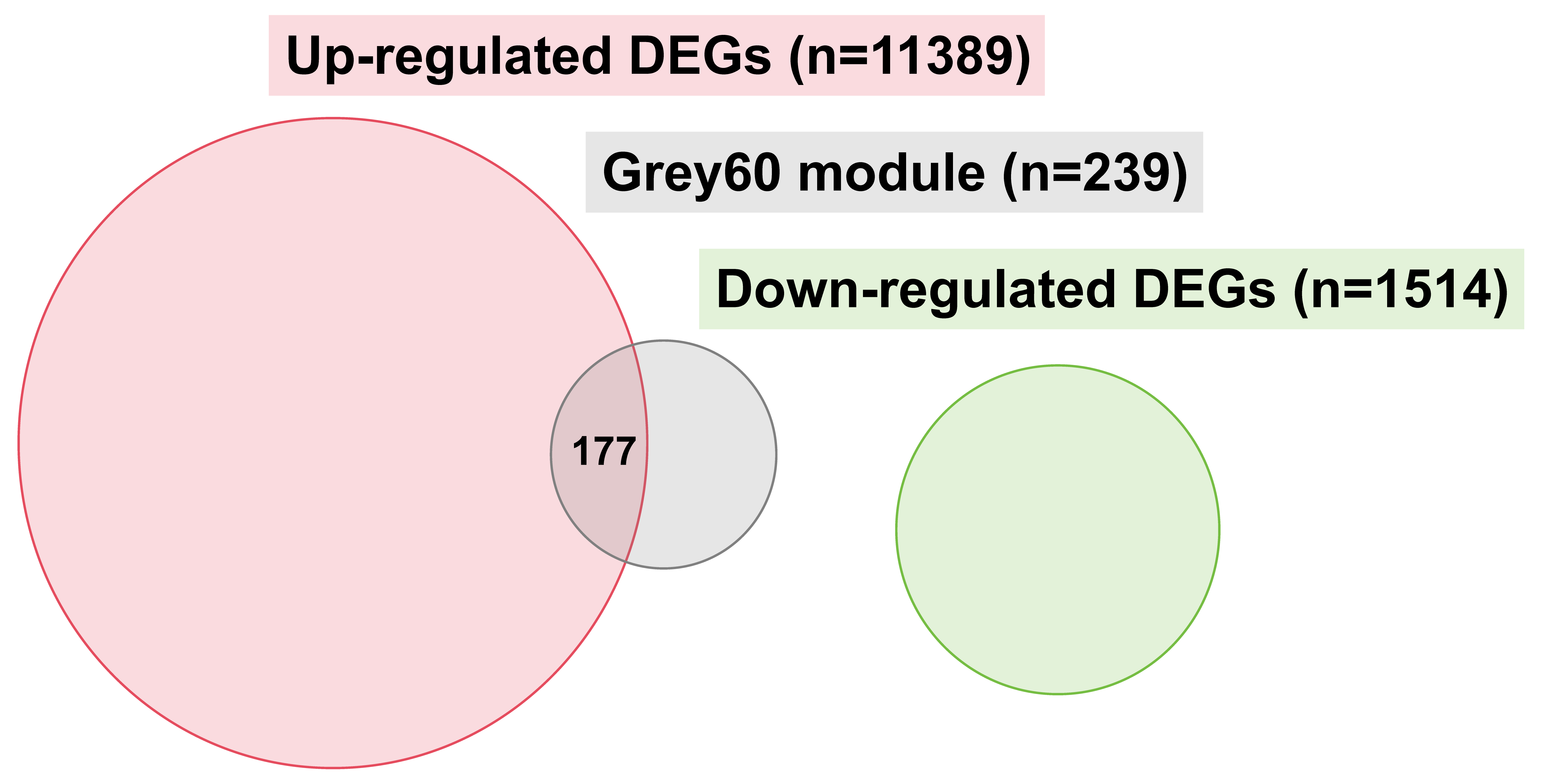


**Supplementary Figure S2. The intersection of DEGs and genes in the WGCNA module most relevant to the high altitude of children with GMPP.**

DEGs, differentially expressed genes; WGCNA, Weighted gene co-expression network analysis; GMPP, general *Mycoplasma pneumoniae* pneumonia.
